# A Comparative Study on Rice Husk, as Agricultural Waste, in the Production of Silica Nanoparticles via Different Methods

**DOI:** 10.3390/ma17061271

**Published:** 2024-03-09

**Authors:** Shengwang Yuan, Yihao Hou, Shun Liu, Yunhai Ma

**Affiliations:** 1 Key Laboratory of Bionic Engineering, Ministry of Education, Jilin University, 5988 Renmin Street, Changchun 130022, China; ysw5968@163.com (S.Y.); shunliu23@mails.jlu.edu.cn (S.L.); 2 Institute of Structured and Architected Materials, Liaoning Academy of Materials, Shenyang 110167, China; houyihao825@163.com

**Keywords:** rice husk, silica nanoparticles, sol–gel methods, calcination, particle size distribution, agricultural wastes

## Abstract

This study explores the conversion of agricultural waste into valuable industrial precursors, specifically focusing on the production of silica nanoparticles from rice husk (RH) via calcination and sol–gel processes. The synthesized particles underwent detailed analysis to assess their chemical composition, structural features, morphological characteristics, and size distribution. This comparative analysis evaluates the effectiveness of various methods in generating silica from RH and examines the impact of different drying techniques, including freeze-drying and conventional thermal drying, on the properties of the resulting silica nanoparticles. Utilizing a combination of sol–gel and freeze-drying techniques produced spherical nanoparticles with diameters of 10 to 20 nm, characterized by size uniformity, clear contours, and minimal aggregation. X-ray diffraction (XRD) analysis identified the amorphous nature of the silica, as evidenced by diffraction peaks typical of amorphous silica in the RH-derived samples processed via different methods. Significantly, the XRD patterns of the calcination-derived silica showed no foreign peaks, indicating a purer amorphous state. The findings of this study are anticipated to contribute to the development of innovative and efficient silica nanomaterials, fostering the sustainable use of agricultural waste.

## 1. Introduction

Research into nanoparticles has seen a significant surge since the late 20th century, a development largely facilitated by advancements in techniques like electron microscopy [1]. Silicon Dioxide Nanoparticles (SiNPs) stand out in this realm, often derived from solid waste, alongside other materials such as carbon-based nanomaterials and metal oxides, including CuO, Ag_2_O, and FeO [2]. The biocompatibility of SiNPs has been confirmed through multiple studies. These nanoparticles are distinguished by their extraordinary properties, which include a remarkable pore and surface structure, extensive surface area, controllable size, uniform dispersion, and robust thermal and mechanical stability. Additionally, their resistance to acid and the simplicity of altering their surface make them even more versatile. It is crucial to note that these findings are grounded in scientific research [3]. The application spectrum of SiNPs is broad, ranging from catalysts, rubber fillers, and electronic coatings to ceramics, concrete, chromatography, anti-corrosion agents, and optical materials [4,5,6].

Diverse methodologies have been developed for SiNP synthesis, incorporating both bottom-up and top-down techniques. Bottom-up approaches, including precipitation, atomic condensation, vapor deposition, laser pyrolysis, sol–gel, and spray pyrolysis, are widely adopted for their precision and control [7,8]. Concurrently, top-down methods such as ball milling, calcination, chemical etching, and sputtering provide alternative pathways [9,10]. Success in achieving desired physicochemical characteristics in SiNPs is largely attributed to the judicious selection of source materials, effective synthesis techniques, optimal condition setting during synthesis, and the implementation of advanced modification or functionalization methods. The sol–gel method, in particular, is renowned for its ability to fine-tune particles’ size, distribution, and shape, primarily due to its systematic control over reaction conditions [11]. The size, shape, dispersion, and aggregation properties of SiNPs are determined by various factors, including pH and thermal conditions, precipitation time, washing and drying techniques, and the use of stabilizers [12].

Literature reviews reveal a variety of techniques for synthesizing SiNPs. Historically, industrial processes have relied on silicates and silanol salts as precursors, but these materials are not only costly and energy-demanding but also contribute significantly to environmental degradation [13,14]. In response, the search for eco-friendly and cost-effective alternatives has gained momentum. Utilizing agricultural waste as a raw material for SiNP synthesis represents a sustainable approach that aligns with energy conservation and environmental protection principles [15]. Agricultural waste is generated during various activities in the agricultural sector. The improper disposal of this waste can lead to increased greenhouse gas emissions, climate change, and threats to global food production, as well as human and environmental health [16,17]. Therefore, it has become increasingly vital to find more viable and environmentally friendly options for managing these wastes [18]. The disposal strategies for agricultural wastes are determined by the physicochemical properties of the wastes themselves. Shichalin et al. [19] utilized rice straw as a precursor to obtain silicon-based materials by spark plasma sintering and subsequently prepared bioceramics that could adsorb 137 Cs radionuclides at 800–1000 °C and 25 MPa.

Rice husk is a prevalent agricultural by-product in many rice-producing regions worldwide [20]. Approximately 20 kg of rice husk waste is produced for every 100 kg of paddy grain processed into rice [21]. The International Grains Council (IGC) has released data that project global rice production to reach 511.3 million tons in 2023/24, resulting in the production of 102.3 million tons of rice husk waste. Rice husk waste is primarily composed of silica, which exists in a highly pure and amorphous form with a large surface area and high reactivity [17]. Rice husk (RH) calcination yields rice husk ash (RHA), predominantly composed of SiO_2_ (around 93%) along with minor quantities of other metal oxides [22]. Given its high silica content, RH has been identified as a viable silica source. Numerous studies have successfully employed RH as a precursor for producing SiNPs of various dimensions [23]. Nassar et al. [24]. extracted SiNPs from RH via a sol–gel method and investigated the effects of titration medium, acid addition order, calcination temperature, and time on SiNP grain size, purity, and yield, demonstrating the importance of these factors in the synthesis process. In their notable work, Liou and Yang implemented a sol–gel method to synthesize SiNPs, optimizing several parameters, including the concentration of the water glass solution, and controlling the aging, gelation reaction temperature, duration, and pH [25]. Nevertheless, they neglected the effect of the different drying methods of the gels on the determination of the particle size of the SiNPs. The drying method significantly affects the size, distribution, and voids of SiNPs, as well as the degree of particle agglomeration.

Calcination presents an alternative method for extracting SiNPs from RH. To fully comprehend the influence of calcination variables such as temperature, duration, and heating rate on silica extraction, detailed kinetic studies are indispensable [26,27]. The specific conditions under which RH is combusted are critical for obtaining reactive silica, which holds the potential to produce high-purity amorphous silica. Kate and Chaurasia [28] indicated that optimal pyrolysis temperatures range from 500 to 700 °C. The purity of the silica obtained was found to increase at a heating temperature of 700 °C for one hour, whereas the silica’s specific surface area diminished as the pyrolysis temperature rose. Additionally, the calcination method has been explored for SiNP extraction from RH. This process involved optimizing the preparation of RH-based silica through calcination, using statistical approaches to refine the procedure [29,30]. The significant factors and optimal processes affecting the particle size distribution of silica extracted from RH by aerobic pyrolysis have been explored. In summary, most researchers have focused their research attention on studying the extraction and purification processes of RH-based silica. Few researchers have conducted longitudinal and cross-sectional comparative studies of the mainstream extraction processes and systematic studies on the particle size and distribution of extracted silica nanoparticles.

In the current study, SiNPs were synthesized, using RH as a precursor, through both calcination (a top-down approach) and sol–gel (a bottom-up approach) methods. This study meticulously characterized the synthesized SiNPs in terms of their particle size distribution, morphology, chemical composition, and structural attributes. It delved into how the different synthesis routes and the drying phase in the sol–gel process impact the characteristics of the SiNPs. The objective of this study is to investigate the optimal method for extracting SiNPs using RH as a precursor and to establish a benchmark for the industrial application of RH as an environmentally friendly silicon source for the production of SiNPs. The findings of this research are crucial for the optimal preparation and quality control of RH-derived SiNPs, as well as for the efficient and high-value application of agricultural waste.

## 2. Materials and Methods

For this research, RH was sourced from a rice mill in Heilongjiang Province, China. The study utilized high-purity anhydrous ethanol, hydrochloric acid, sodium hydroxide, and acetone, all procured from Beijing Chemical Reagent Co., Ltd., Beijing, China. To prepare the RH for experimentation, a blast dryer from Jilin Ji Da Electromechanical Equipment, Changchun, China, was used to dry the samples at a consistent temperature of 105 ± 5 °C for 24 h. After the drying process, the RH samples were packaged and preserved in a desiccator for later use. The proximate analysis of these samples was conducted in accordance with ASTM standards. The preliminary and final analytical results of the samples are presented in Table 1.

In our earlier research, we concentrated on developing a method for creating silica from RH using a top-down approach, specifically calcination. The process was refined to a specific protocol, identified as 270/700 ZW [29,30]. Initially, RH was boiled in deionized water continuously for 2.5 h. After boiling, it underwent a triple rinse with deionized water and was drained to eliminate contaminants. The purified RH was then dried in a microwave oven for 7 min at 450 W, in batches of 50 g each. Following microwave drying, 15 g of RH was placed in a muffle furnace crucible (Best Equipment, Shanghai, China) for roasting and calcining, thereby producing RHA. The calcination involved a two-step heating process: first at 270 °C for 4 min and then at 700 °C for 2 h, with a heating rate of 10 °C/min and a cooling rate of 20 °C/min. The final product was subjected to milling in a planetary ball mill (Ten Can Powder, Shenzhen, China), with specific parameters including a feed rate of 10 g, a speed of 300 rpm, an intermittent milling time of 10 min, and a total milling duration of 4 h. This procedure for obtaining RHA-SiO_2_ was designated as ‘RH-T’.

In this detailed study, the sol–gel method, an innovative bottom-up approach, was employed for the synthesis of silica from RH. The process began with treating 100 g of RH with 1000 mL of a 2 M HCl solution at a maintained temperature of 90 °C for three hours, ensuring thorough interaction. Following this acid treatment, the RH was subjected to extensive washing with deionized water to obtain a constant neutral pH of 7, indicating the removal of residual acidic components. For effective separation of the treated RH from the liquid, a vacuum pump (Autoscience, Tianjin, China) and Whatman no. 41 filter paper were utilized, ensuring a precise filtration process. After filtration, the RH underwent a crucial treatment with a 2 M NaOH solution, facilitating the solubilization of silica and resulting in the formation of sodium silicate solutions (SSSs) [28]. This SSS was then boiled for an extended period of two hours and subsequently cooled to ambient temperature, allowing for the separation of solid residues. The cooling process was followed by a meticulous filtration using the same vacuum setup, removing any undissolved particles. The pH of the SSS was then carefully adjusted to neutrality using hydrochloric acid, creating a conducive environment for gel formation. However, to avoid the formation of a gel and maintain a liquid state, the solution was constantly stirred while being treated with a dilute sulfuric acid solution, adjusting the pH to a highly acidic range of 1–2. The resulting neutralized liquid was then centrifuged, isolating the silica particles efficiently. Post centrifugation, the silica particles were thoroughly washed with deionized water to eliminate any remaining impurities, specifically targeting sodium chloride (NaCl). To explore the impact of various drying methods on the silica particles’ properties, the sample was divided into two equal portions. The first portion underwent a low-temperature drying process in a vacuum freeze dryer (SCIENTZ, Ningbo, China) at −60 °C for 24 h, yielding a variant of silica named RH-S1. In contrast, the second portion was subjected to a higher temperature drying regime in a blast drying oven (Jilin Ji Da Electromechanical Equipment, Changchun, China) at 105 ± 5 °C for the same duration, resulting in another variant of silica, named RH-S2. This comparative analysis of drying methods was crucial for assessing their influence on the morphological and size distribution characteristics of the silica particles.

For each method of silica preparation, a 0.5 g sample was carefully placed into a centrifugal tube. To this, 10 mL of pure ethyl alcohol was added. To ensure the uniform distribution of particles, each tube was subjected to ultrasonic agitation for 10 min. Following this, particle size analysis was conducted. The average particle size, represented as D50 in nanometers, and the PDI of the SiNPs were determined using DLS technology. This analysis was performed using a Malvern Zetasizer (Malvern Instruments, Moorburn, UK), which was calibrated to maintain a constant temperature of 25 °C. For accuracy and reliability, each silica sample’s measurement was replicated three times. To avoid any potential cross-contamination between samples, the testing chamber was meticulously cleaned with acetone after each analysis.

The silica’s structure was analyzed using a Rigaku D/max-2500 XRD device, which scanned the samples from 10° to 80° over abroad range of 2θ angles with CuKα radiation at 40 kV and 30 mA. To interpret and document the intricate crystalline patterns of the silica samples, we employed the sophisticated EV A™ software (PDXL, Bruker, Karlsruhe, Germany), renowned for its precision in structural analysis.

To precisely characterize the chemical composition and functional groups of the silica, we utilized the advanced FTIR system from Nicolet Thermo, Erlangen, Germany. Before analysis, to ensure the accuracy of the FTIR spectra, each silica sample was thoroughly dried in an oven for over 10 h. This process effectively removed any surface-adsorbed moisture that could otherwise obscure the spectral data. The sample preparation was conducted under a controlled environment provided by a heat-preservation heating lamp. The FTIR scanning protocol involved a meticulous process with a scanning rate of 5 cm^−1^/s across a comprehensive wavelength range of 400 to 4000 cm^−1^, thus capturing a detailed spectral footprint of the silica.

Following the meticulous process of ball milling, we examined the silica nanoparticles’ morphology in depth using the high-resolution Zeiss EVO 18 SEM (MERLIN, Wiefelstede, Germany), operated in a low-vacuum environment. The non-conductive nature of the silica samples necessitated the application of a thin, conductive gold layer on each sample. This coating was critical for enhancing their electrical conductivity, thereby enabling a clear and detailed visualization of the nanoparticles’ structure during the SEM analysis.

## 3. Results and Discussion

**Figure 1:**
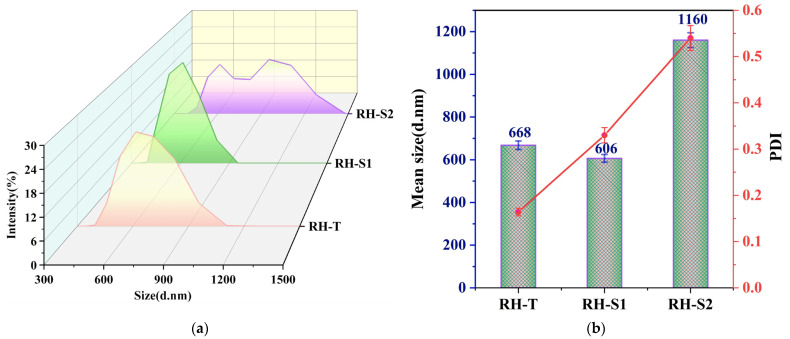
(**a**) Typical particle size distribution pattern of silica from RH after different treatment methods. (**b**) Average size and PDI of silica particles.

Figure 1a presents a comparative analysis of the particle size distributions of silica synthesized via different methodologies. The graphical representation demonstrates variations in peak height and width across the different silica samples. Specifically, RH-S1 is characterized by the most pronounced peak, indicating a narrow distribution, with its peak apex situated around 600 nm. In contrast, RH-T displays a distinct single-peak profile, with a broader distribution spanning 400 nm to 1100 nm and an intermediate peak height between RH-S1 and RH-S2. RH-S2, on the other hand, showcases a bimodal distribution pattern, extending from 400 nm to 1500 nm, and exhibits the least pronounced peak. Based on these observations, it can be inferred that the particle size distribution range of the silica samples follows the order RH-S2 > RH-T > RH-S1, with RH-S2 having the widest distribution.

Figure 1b presents a comparison of silica particle sizes after various treatment procedures. It illustrates that the average dimensions of these particles are organized in descending order: RH-S2 (606 nm) surpasses RH-T (668 nm), which in turn is larger than RH-S1 (1160 nm). This order aligns with the broad range of particle size distribution observed in Figure 1a. The observed variation in sizes can likely be attributed to the distinct methodologies employed in the freeze-drying technique. This technique gently removes water and other solvents through their sublimation in a vacuum environment. Such a process minimizes capillary forces and thermal stress, consequently leading to negligible shrinkage. Additionally, it results in particles with an elevated specific surface area and considerable porosity. On the other hand, the heat drying method tends to diminish the gel’s volume. The rapid expulsion of liquid from the silica’s pores during this process induces blister formation, along with the shrinkage and wrinkling of the silica gel. The freeze-drying technique, conversely, preserves the material’s structure, avoiding any contraction in the gel matrix [24].

PDI, as a dimensionless indicator derived from the autocorrelation function, offers insights into particle size variance based on DLS data [11]. A PDI ranging from 0.1 to 0.5 typically denotes accurate measurements and stable colloidal formations. A PDI close to 0.7, however, suggests a potential uneven distribution within the sample, possibly containing larger particles or aggregates with a broad size range. As shown in Figure 1b, RH-T demonstrates the lowest PDI, followed by RH-S1, and then RH-S2 with the highest. These values, spanning from 0.15 to 0.55, indicate the reliability of our measurements and well-formed colloidal suspensions. Specifically, the silica dispersion achieves the greatest uniformity in the RH-T suspension, whereas it appears the least consistent in the RH-S2 suspension. The reason for this may be that the aerobic calcination method removes a significant amount of water from the surface of SiNPs, resulting in a reduction in the hydrophilic groups on the surface and a decrease in the agglomeration phenomenon of SiNPs in anhydrous ethanol. In contrast, RH-S2 used the oven-drying method to obtain SiNPs with more surface hydrophilic groups, which led to more serious inter-particle agglomeration.

**Figure 2:**
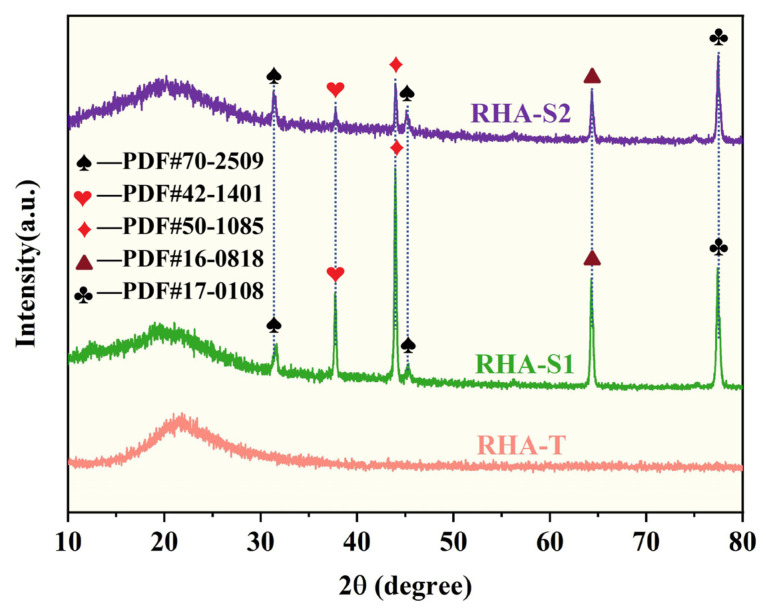
The X-ray diffraction patterns of the silica from RH after different treatment methods. (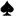
—NaCl, 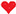
—Cristobalite, 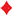
—Graphite, 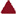
—Na_2_SiO_3_, 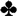
—NaC64.)

Presented in Figure 2 are the XRD patterns for silica derived from RH using various processing techniques. These patterns depict a diffuse broadening in the range of 2θ = 18° to 35° across the XRD traces, which is a hallmark of the presence of amorphous SiO_2_ [31]. The absence of sharp, distinct peaks that are typically indicative of non-crystalline SiO_2_ reaffirms the amorphous status of the silica [32]. Moreover, the XRD pattern of RH-T, produced through a top-down approach like calcination, shows an absence of significant peaks, suggesting an amorphous structure with minimal impurity. In contrast, the silica from RH prepared via a bottom-up method (sol–gel) displays pronounced X-ray diffraction peaks. Notably, in the XRD spectra of RH-S1 and RH-S2, a subtle rise is observed at 2θ values between 18° and 30°, along with minor crystalline inclusions such as NaCl (PDF#70-2509), Cristobalite (SiO_2_, PDF#42-1401), Carbon (C, PDF#79-1467), Na_2_SiO_3_ (PDF#70-2509), and NaC₆₄ (PDF#17-0108). Both the RH-S1 and RH-S2 samples display NaCl diffraction peaks, possibly due to the strong adsorption of Na^+^ and Cl^−^ on the silicon surface during silicate gel formation, leading to their entrapment within the silicon matrix and resistance to removal [33]. Acid pretreatment modifies the chemical composition of RH-derived products without altering their structure. Silica’s disordered structure becomes more ordered when subjected to high-temperature heating [34]. The Na_2_SiO_3_ peak emerges due to the gel encapsulating the solution during gelation, resulting in uneven mixing and subsequent drying that leads to the precipitation of sodium silicate particles. This study utilized RH, after hydrochloric acid decontamination, as the precursor of silica, which was then extracted using the sol–gel method. RH initially contained a significant amount of carbon, which was relatively stable and did not react with hydrochloric acid and sodium hydroxide. However, some of the carbon could not be completely removed by washing and filtration, resulting in its deposition on the surface of the silica, where it was detected. The detection of the NaC64 diffraction peak, an alkali metal–graphite intermediate, is a novel aspect that merits further examination.

**Figure 3:**
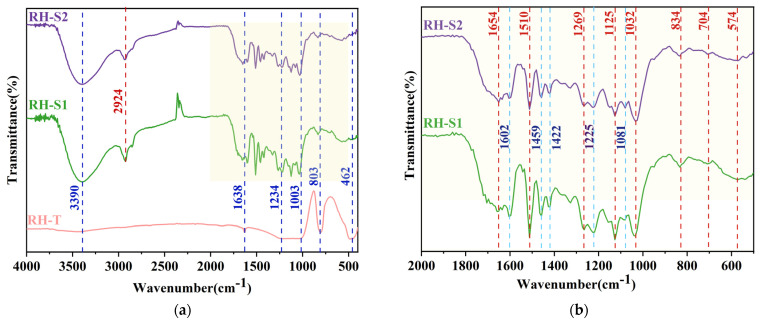
FTIR spectra of RH-based silica (**a**) and spectral spectra of local features (**b**) obtained via different processes.

Figure 3a delineates the FTIR spectra of the silica derived from RH through various methods. The RH-T sample exhibits a broad spectral band ranging from 2500 to 3600 cm^−1^, indicative of O-H stretching vibrations in the RH-based silica. This band confirms the hydroxyl groups on the surface of the silica nanoparticles and the water absorption in the samples [24,35]. In all three samples’ FTIR spectra, the vibrational signature at 1003 cm^−1^ aligns with the asymmetric stretching vibrations of Si-O-Si bonds [36]. The occurrence of peaks at 803 cm^−1^ and 462 cm^−1^ is representative of the symmetric stretching and asymmetric bending vibrations of Si-O-Si bonds, respectively, establishing the presence of SiO_2_ [37,38]. The presence of these amorphous silica characteristic groups proves that there is an amorphous silica component in all the samples. Additionally, the vibrational peaks around 3400 cm^−1^ and 1640 cm^−1^ are ascribed to chemically bound water, represented by O–H and H–O–H bond vibrations [39,40].

In Figure 3b, the enhanced FTIR spectra of the RH-S1 and RH-S2 samples are displayed, highlighting their particular absorption peaks. The subtle peak observed between 3000 and 2800 cm^−1^ is associated with the C–H stretching vibrations in alkanes [41]. Near 1654 cm^−1^, a faint absorption peak corresponds to C=C stretching vibrations [42]. The absorption bands found between 1600 and 1450 cm^−1^ are linked to C=C backbone vibrations, and the peak around 1422 cm^−1^ is connected to C-N stretching vibrations [43]. The functional groups C-H, C=C, and C-N maybe produced by the acid degradation and alkali degradation reactions of the cellulose, lignin, and hemicellulose in RH in the sol–gel method. The most pronounced transmittance peaks in the geopolymerized mixture, typically at 1269 cm^−1^ and 1225 cm^−1^, arise from the asymmetric stretching vibrations of the Si–O-T band, where T signifies tetrahedral Si or Al [44]. Peaks at 1125 cm^−1^, 1081 cm^−1^, and 1032 cm^−1^ are indicative of Si-O-Si asymmetric stretching [45,46]. These peaks are characteristic of crystalline silica, indicating that the silica extracted from RH via the sol–gel method contains a component of crystalline silica, confirming the results in the XRD diffractograms. Additionally, the absorption band at 834 cm^−1^ is indicative of C-Si stretching vibrations within the silicon carbide crystal structure [47]. Overall, the FTIR absorption peak positions for RH-S1 and RH-S2 remain consistent, albeit with variations in peak intensity, suggesting that the drying method employed in the sol–gel process minimally impacts the types of functional groups present [48,49]. However, the SiNPs prepared from rice husks using the sol–gel method contained more impurities than those prepared using the aerobic calcination method. This conclusion is also supported by their XRD patterns.

**Figure 4:**
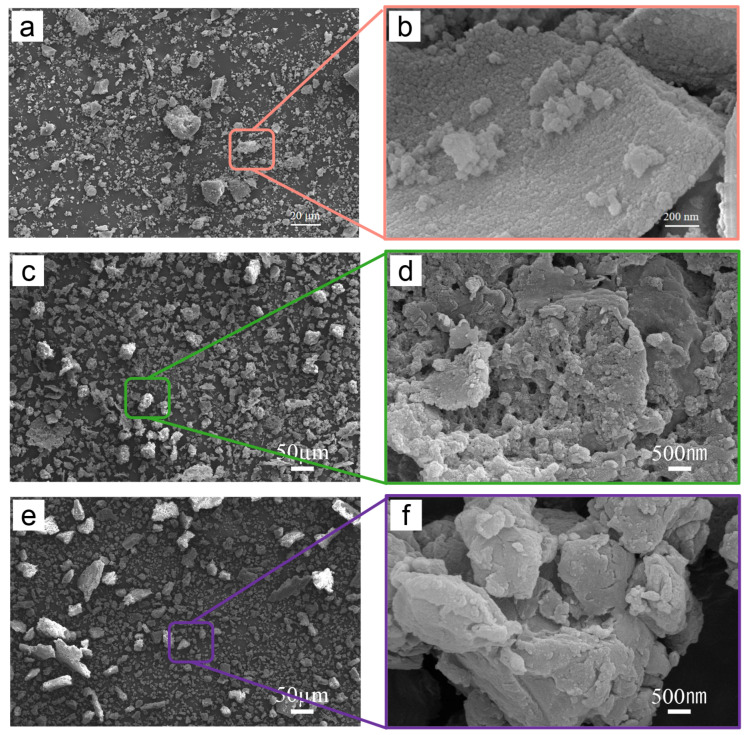
SEM overview images of a selection of silica particles obtained via different methods of RH treatment, showing the microforms of the RH-T sample obtained by calcination (**a**), local magnified morphology of the RH-T sample (**b**); microforms of the samples RH-S1 (**c**) and RH-S2 (**e**), obtained via the sol–gel method, and local magnified morphology of samples RH-S1 (**d**) and RH-S1 (**f**).

Figure 4a,b illustrate the microstructural characteristics of silica particles synthesized via the top-down approach (calcination) using RH. The silica labeled RH-T, post-calcination, manifests a predominantly spherical shape. Observations at higher magnifications reveal a rough exterior surface and clusters of nanospheres (as seen in Figure 4a,b). Within these clusters, pores form interconnected slits, which are evident in Figure 4b. The scanning electron microscopy (SEM) images (Figure 4c,d) depict a composition of irregularly sized agglomerates and spherical particles interspersed within a porous network. These spherical particles maintain a relatively consistent size, ranging from 10 nm to 20 nm, and their agglomeration contributes to the surface’s roughness. The SEM micrograph in Figure 4e shows RH-S2 particles exhibiting irregular flake-like or lump-like structures, varying from 10 µm to 150 µm in size, possibly due to the limited duration of manual milling. In Figure 4f, at a magnification of 50,000×, the SEM image of the RH-S2 silica reveals layers of clustered nanoparticles with smooth surfaces, obscuring individual particle identification. The boundaries of these silica nanoparticles are indistinct, as they exist in an aggregated and amorphous state [50]. The surface of the silica nanoparticles prepared in this study is rich in hydrophilic groups (-OH), as confirmed by Fourier transform infrared spectroscopy analysis. Oven drying causes moisture aggregation and a large amount of water vapor, resulting in the silica nanoparticles being in constant contact with numerous water molecules. The agglomeration phenomenon among silica nanoparticles is intensified by the strong inter-particle interactions and capillary forces generated by water evaporation. As a result, the average particle size dimension increases and the particle size distribution becomes uneven. Moreover, vacuum freeze-drying freezes water directly into ice at low temperatures (−60 °C), reducing the activity of water molecules. This intensifies the sublimation phenomenon in the low-temperature vacuum environment, while the overflow water vapor is quickly removed by the dehumidification system. This results in a decrease in the chance of silica nanoparticles interacting with water molecules, which inhibits inter-particle agglomeration. Additionally, a significant number of pore structures are obtained after the sublimation of ice crystals.

## 4. Conclusions

Exploring different methodologies to produce amorphous silica from RH highlights a sustainable and eco-friendly approach to transforming agricultural waste into valuable nanomaterials. This research uniquely investigates the impact of both top-down and bottom-up processes, represented by the calcination and sol–gel methods, on the generation of green silica nanoparticles. Utilizing RH as the precursor, this study primarily focuses on the nanoparticles’ size distribution. The smallest mean particle size, recorded as 606 nm, was achieved through the sol–gel process combined with freeze-drying. Conversely, the largest mean particle size, measured as 1160 nm, resulted from the sol–gel process followed by heated drying. Freeze-drying’s ability to maintain the material’s structure and reduce nanoparticle aggregation was established. The gel’s integrity remained intact without shrinkage, leading to decreased nanoparticle clumping. A further analysis of X-ray diffraction patterns revealed that all RH-derived silica samples contained diffraction peaks characteristic of amorphous silica, affirming their amorphous nature. The silica produced through calcination showed no heterogeneous peaks, indicating its high purity. The FTIR spectra of these nanoparticles displayed absorption bands confirming the presence of silica characteristics. However, the sol–gel method is unable to fully eliminate the residual organic matter in silica nanoparticles, resulting in the appearance of a crystalline structure in the silica. These factors contribute to a decrease in the purity of the silica nanoparticles obtained through this method. SEM imaging provided insights into the diverse morphological structures of the samples, showcasing different degrees of agglomeration. Notably, spherical silica particles synthesized via the sol–gel method coupled with freeze-drying demonstrated uniformity and well-defined shapes, with sizes ranging from 10 nm to 20 nm and minimal agglomeration. This shows that the drying process has a considerable impact on the particle size, distribution range, and morphology of the silica nanoparticles extracted from RH through the sol–gel method. These findings underscore the efficacy of using agricultural waste as an environmentally friendly and cost-effective precursor for producing sustainable nano-silica.

## Figures and Tables

**Table 1:** Proximate and ultimate analyses of RH samples.

Name	Proximate Analysis (wt %)			Ultimate Analysis (wt %)				
	V	A	FC	C	H	N	S	O
RH	59.6	24.8	15.6	43.5	6.4	0.6	0.1	35.2

Note: The table displays the volatile content (V), ash content (A), and fixed carbon (FC) of the sample. Additionally, it shows the percentages of carbon (C), hydrogen (H), nitrogen (N), sulfur (S), and oxygen (O) in the sample.

## Data Availability

Data are contained within the article.
